# Use of artificial intelligence on Electroencephalogram (EEG) waveforms to predict failure in early school grades in children from a rural cohort in Pakistan

**DOI:** 10.1371/journal.pone.0246236

**Published:** 2021-02-08

**Authors:** Muneera A. Rasheed, Prem Chand, Saad Ahmed, Hamza Sharif, Zahra Hoodbhoy, Ayat Siddiqui, Babar S. Hasan

**Affiliations:** 1 Department of Paediatrics & Child Health, Aga Khan University, Karachi, Pakistan; 2 Department of Artificial Intelligence, Ephlux Pvt. Limited, Karachi, Pakistan; Gachon University Gil Medical Center, REPUBLIC OF KOREA

## Abstract

Universal primary education is critical for individual academic growth and overall adult productivity of nations. Estimates indicate that 25% of 59 million primary age out of school children drop out and early grade failure is one of the factors. An objective and feasible screening measure to identify at-risk children in the early grades can help to design appropriate interventions. The objective of this study was to use a Machine Learning algorithm to evaluate the power of Electroencephalogram (EEG) data collected at age 4 in predicting academic achievement at age 8 among rural children in Pakistan. Demographic and EEG data from 96 children of a cohort along with their academic achievement in grade 1–2 measured using an academic achievement test of Math and language at the age of 7–8 years was used to develop the machine learning algorithm. K- Nearest Neighbor (KNN) classifier was used on different model combinations of EEG, sociodemographic and home environment variables. KNN model was evaluated using 5 Stratified Folds based on the sensitivity and specificity. In the current dataset, 55% and 74% failed in the mathematics and language test respectively. On testing data across each fold, the mean sensitivity and specificity was calculated. Sensitivity was similar when EEG variables were combined with sociodemographic, and home environment (Math = 58.7%, Language = 66.3%) variables but specificity improved (Math = 43.4% to 50.6% and Language = 32% to 60%). The model requires further validation for EEG to be used as a screening measure with adequate sensitivity and specificity to identify children in their preschool age who may be at high risk of failure in early grades.

## Introduction

Universal primary education is regarded as the key to the successful development and prosperity of future generations. United Nations Education, Scientific and Cultural Organization (UNESCO) states that literacy skills are fundamental to informed decision-making, personal empowerment, and participation in the local and global social community [1]. Hence, UNESCO’s goal in the post Millennium Developmental Goals era was to “ensure equitable and inclusive quality education and lifelong learning for all” [1] and now the Sustainable Development Goal 4 aims to achieve this outcome by ensuring that all girls and boys complete free, equitable and quality primary and secondary education by the year 2030 [2].

A report in 2013 found that globally 59 million children of primary-school age (6 to 11 years of age) were out of school [3]. Estimates suggest that 1 in 5 of those children had dropped out and 2 in 5 of dropped-out children never set foot in school again. Of these, 50% (33 million) live in sub-Saharan Africa followed by Central and Southern Asia which has the second highest number of out-of-school children (11 million) [3]. Pakistan is among the top five countries which are home to more than one-third of all out-of-school children [3]. Of the estimated total primary school-going population of ages 5–9 years (approximately 21.4 million,), 68.5% are enrolled in school, with a preponderance of boys (56%) as compared to girls (44%) [4]. Further, only 66.8% of these children continue schooling till grade five and 33.2% drop out before completing primary education [4]. Overall education indicators in Pakistan are not very encouraging either. According to Pakistan Demographic and Health Survey 2017–18, 49% of ever-married women and 25% of ever-married men have no education while 16.5% of women and 20.3% of males have completed primary education [5].

Causes of not achieving primary education include, but are not limited to, the lack of available school facilities, the inability to afford an education, the lack of importance given to education and hence the unwillingness to enroll in or continue with primary education and lack of school readiness in children [6]. Health concerns such as problems related to hearing and vision and psychological factors have been found to be more prevalent in students who failed a grade in school hence affecting their readiness for school [7]. Among psychosocial factors, poor executive function and lower cognitive ability, which could be due to a multitude of factors such as malnutrition and recurrent illnesses may affect children’s school readiness leading to poor academic achievement in primary grades [8]. Additionally, academic struggle in the early years of education may be indicative of short-term behavioral problems and predictive of school dropout [9].

While the importance of education and high number of children at risk of not completing primary education has been taken note of by the system, a challenge arises in early identification of these children. Early screening for at-risk children can help professionals in designing appropriate interventions to ensure greater success rate in early grades of school [10]. In the past, mechanisms for identification of at-risk children included hearing and visual tests, or direct cognitive assessment of children [11]. Accurate cognitive testing, as a screening tool, is time consuming, expensive and requires higher level professional training which may not always be feasible in resource constrained settings [11].

Recently, the use of the Electroencephalogram (EEG), a test used to record electrical activity in the brain, has been observed in more than just traditional neurological indications. Thatcher et al reported that EEG has a discriminant ability of more than 90% in identifying individuals with high and low IQ [12]. Further, a study conducted in rural Pakistan reported that children with better cognitive skills and increased executive function had an increase in gamma frequency bands as opposed to those with lower cognitive skills [13]. Tarullo et al. (2017) reported that EEG can detect differences in a child’s brain maturity and executive functioning at an earlier age than traditional testing [13]. EEG can be a more objective measure for screening as it is less affected by motivation compared to psychological testing. In high-income countries EEG is relatively cheap. Experts have suggested that quantitative EEG (which provides digital displays of EEG unlike paper-EEG) may be one of the solutions to meet demands of EEG in inefficient healthcare systems due to flexible way of recording, reviewing and storing data [14]. However, a major hurdle in the use of EEG in developing countries, which also have the greater burden of out of school children, is that it is resource intensive and requires highly trained personnel for interpretation, thereby limiting its use for mass screening. However, automating interpretation of these waves using artificial intelligence can help overcome the hurdle of lack of trained individuals and increase availability of EEG based screening in such contexts.

Machine-learning (ML), a more defined subset under the umbrella of Artificial Intelligence (AI), is an effective tool for the efficient analysis of large sets of data, especially those in which there are recognizable patterns [15] which has also been documented to be advantageous for analysis of EEG signals due to smaller probability of bias and high sensitivity to pattern recognition [16]. Several studies have used ML models on existing performance scores of students to predict students school performance with an accuracy of 70–80% and dropout with an accuracy of 63–83% [17,18].

Combining the reduced probability of human bias when recording EEG signals with the ability of a ML algorithm to classify data provides a new avenue with which children at-risk of failure in early grades may be identified. The objective of this study was to determine the potential of a ML algorithm to evaluate the power of EEG data collected at age 4 in predicting academic achievement (Math and Language) at age 8 among rural children in Pakistan.

## Materials and methods

### Study setting

The study sample included 219 children whose EEG data were collected to examine the links between EEG gamma power with cognition at age 4 years in an earlier study [13]. These 219 children were a sub-sample of a large trial birth cohort (n = 1489) exposed to early interventions between birth to 2 years of age [19]. The sub-sample had been randomly selected from the full cohort, stratified by the respective intervention groups at a prospective follow-up at age 4 years [13]. In the previous study, EEG at 4 years was associated with executive functioning at the same age and in the current study we sought to examine if similar association continued with academic achievement at age 8 years. An analysis of the sample included indicated no significant differences on the academic achievement scores when compared to the full sample at 4 years. The included sample had slightly higher IQ (*t* = 6.1, *p* = 0.013) and was younger (*t* = 6, *p* = 0.014) compared to the rest of the sample. EEG was recorded using a 64-channel high-density Geodesic sensor net (Electrical Geodesics, Inc.; Eugene, OR) and a Net Amps 300 high input amplifier. Continuous EEG was recorded for four blocks of one minute each with a total of four minutes. A central fixation cross was presented on a gray background and a brief silent video with bubbles popping up played between blocks to keep the child engaged [13].

### Data collection

Detailed procedures of the data collected at 4 years (sociodemographic, intelligence test scores and EEG) have been described in the paper published earlier [13]. The demographic data measures included questions regarding household income, number of family members, parental education, occupation, household assets and food insecurity. Intelligence test scores were assessed using an adapted version of Wechsler Preschool and Primary Scales of Intelligence (WPPSI) III while home environment was measured using the Home Observation for Measurement of Environment (HOME) Inventory. EEG data were collected by trained research associates. Trained community-based teams obtained data at 8 years of age. The academic achievement was measured at 8 years using academic achievement test developed according to framework by USAID for measurement of Mathematics [20] and Language [21]. The test items were aligned with provincial curriculum for grade 2 and had sections on English, Mathematics, and local language (*Sindhi*). For the current analysis, we included only Mathematics and local language. English was excluded as it is not the mother-tongue of the participants and may have additional factors contributing toward failure on the test. Children attaining scores of at least 40% were considered to have passed this test. Ethics approval was obtained from the Ethics Review Committee of the Aga Khan University in Pakistan. The primary caregivers provided written consent (or thumb impression) for the assessments at 8 years and could withdraw from the study anytime. The data received for analysis were fully anonymized.

### EEG feature extraction

The 64-channel EEG data were used at the input source and exported in European Data Format (edf). Data were sampled at 500Hz, one second per segment. Out of 64-channels, the data of 6 channels i.e. frontal (6, 12, 60) and parietal (28, 34, 42) were selected based on literature available for EEG waveforms in young children [13] and used for preprocessing. The EEG signal was four minutes long consisting of four baseline events. The start time of each event was identified. Each baseline event was of 60 seconds in length. The first baseline event was selected for further analysis as it was present in the majority of the recorded EEG data files. For the selected EEG baseline event, the 60 seconds epoch data were analyzed and any recording with bad channel data within the selected channels and epoch was discarded. Afterward the data was segmented into an array and the power spectral density (PSD) was calculated using the Welch method [22]. The length of the segment was 1 second and the “Hann” window was used as an appropriate size of overlap. After calculating the PSD, the mean PSD between the gamma brainwave frequency (21-45Hz) was extracted for all 6 channels and its log transformed values were used as a feature in the machine learning algorithm. Feature extraction and preprocessing of EEG was done using the MNE library [23] available in python [24]. Python’s SciPy library [25] was used to calculate the PSD using the Welch method.

### Proposed machine learning methodology

Classification is the method of identifying patterns/learning concepts from a dataset and predicting the label/class of the dataset. For predicting the performance, classification was conducted separately for outcomes in mathematics and language based on the following model combinations i) EEG features, ii) IQ feature, iii) Sociodemographic (socioeconomic status, household food security, parent education and parent occupation), home environment and IQ features combined iv) Sociodemographic, home environment and EEG features. We sought to examine the predictive power with and without sociodemographic and home environment variables and also compare the predictive power with traditional IQ testing scores.

We used K Nearest Neighbors (KNN) for classification. KNN is a non-parametric technique used to classify new instances based on the similarity (distance metric) with its neighbors [26,27]. The KNN classifier was trained using the default parameters as defined in the scikit-learn implementation of the algorithm [28]. The default settings are *n_neighbors = 5*, *weights = ’uniform’*, *leaf_size = 30*, *p = 2*, *metric = ’minkowski’*. The parameter details can be reviewed from the official documentation [28].

### Validation methods

**Fig 1:**
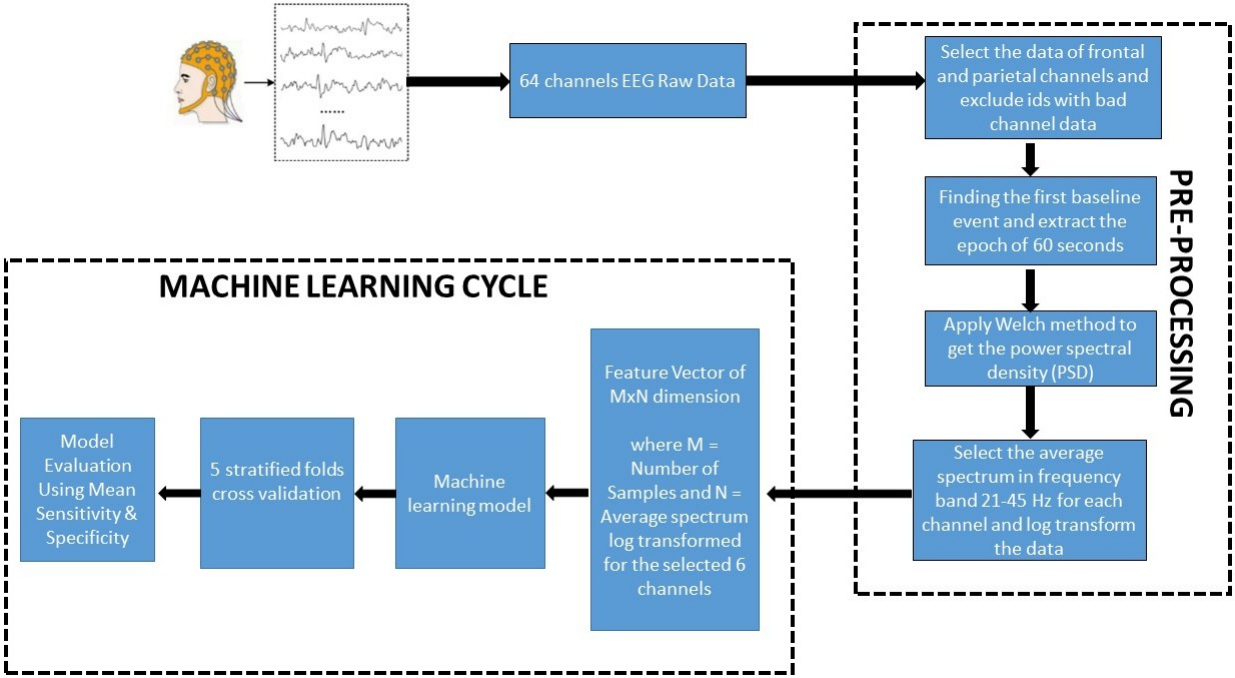
Framework of pre-processing and classification.

Supervised machine learning techniques require a considerable amount of data to learn meaningful relationships and validate the results. However, when the dataset is small and imbalanced it requires advanced techniques such as resampling the data to model the available features for extracting useful insights. Also, a simple train-test split in such cases may not provide an accurate understanding of the model performance. Hence to overcome this problem we used 5 Stratified Folds [29] to validate the performance of our machine learning classifier across different distributions of the data. For each fold Synthetic Minority Oversampling Technique (SMOTE) [30] was applied on the training data to balance the classes. SMOTE is used to avoid over fitting of the ML model on skewed classes. The performance of the model trained across each fold was then tested on the remaining unseen data for that fold. The performance metrics used were sensitivity and specificity. For all these metrics the average of testing data for each fold was reported. Fig 1 describes the process of our implemented methodology.

### Statistical analysis

Data were examined for normality prior to analysis. Data on the demographic characteristics of the study participants was reported as percentages and mean +/- standard deviations or frequencies as appropriate. To test the difference between participants by the outcomes, t- test was applied for continuous variables and chi-square was used for categorical data adjusted using Fisher exact test. Table 1 indicates the two metrics used to evaluate the performance of machine learning algorithms.

**Table 1:** Metrics used to evaluate performance of machine learning.

Metric	Explanation	Formula
Sensitivity	It is the ratio of correctly predicted positive samples to all the samples of the class of interest (failure).	$Sensitivity=\frac{tp}{tp+fn}$
Specificity	It is the ratio of correctly predicted negative samples to all the samples of the class not of interest (pass).	$Specificity=\frac{tn}{tn+fp}$

Note: *t*_*p*_, *f*_*p*_, *t*_*n*_ *and f*_*n*_ represents true positive, false positive, true negative and false negative respectively.

## Results

Out of 219 children, 96 tracings were used in the ML model. Reasons for exclusion included lost to follow-up at age 8 years (n = 11), data not available due to corrupt EEG files, or no data for baseline event 1 (n = 34) and bad channel data (n = 78). The analysis of the outcomes of final sample of 96 children indicated that children had poorer performance on the language test with ~74% failing the test compared to 55% on test of mathematics test. Demographically from the final sample of 96 children, 66.7% of mothers and 24% of fathers were illiterate. Mothers were predominantly housewives (74%) and fathers mainly were skilled workers (75.3%). Demographics and academic performance characteristics at 7 to 8-year follow-up are shown in Table 2. The table shows the difference between the groups (either passed or failed on the mathematics and language achievement test) based on their sociodemographic characteristics. There were significant differences between both groups on paternal education (χ^2^ = 4.3, p = 0.039 for Math and (χ^2^ = 7.4, p = 0.006 for language), maternal education (χ^2^ = 11.1, p = 0.001 for Math; (χ^2^ = 10.8, p = 0.001 for language), socioeconomic status (χ^2^ = 23.8, p<0.000 for Math; χ^2^ = 15.8, p = <0.003 for language), household food security (χ^2^ = 8.7, p<0.003 for Math; χ^2^ = 7.3, p = <0.009 for language) and home environment (*t* = 8.9, p<0.004 for Math; *t* = 22.6, p = <0.000 for language). IQ scores were significantly different for the group who passed or failed language test (χ^2^ = 10.0, p = 0.002).

**Table 2:** Sociodemographic characteristics of the study participants.

Variables	Passed the Math test (n = 43)	Failed the Math test (n = 53)	t/chi	P value	Passed the language test (n = 25)	Failed the language test (n = 71)	t/chi	P value	Total
Age (years)(Mean, SD)	7.23 (0.43)	7.32 (0.47)	0.903	0.344	7.3 (0.46)	7.3 (0.45)	0	0.987	7.3 (0.45)
Gender									
Male	22 (51.2)	31 (58.5)	0.515	0.473	12 (48.0)	41 (57.7)	0.71	0.399	53 (55.2)
Female	21 (48.8)	22 (41.5)			13 (52.0)	30 (42.3)			43 (44.8)
Father’s education									
Illiterate	6 (14.0)	17 (32.1)	4.28	0.039	1 (4.0)	22 (31.0)	7.4	0.006	23 (24.0)
Literate	37 (86.0)	36 (67.9)			24 (96.0)	49 (69.0)			73 (76.0)
Mother’s education									
Illiterate	21 (48.8)	43 (81.1)	11.14	0.001	10 (40.0)	54 (76.1)	10.82	0.001	64 (66.7)
Literate	22 (51.2)	10 (18.9)			15 (60.0)	17 (23.9)			32 (33.3)
Father’s occupation									
Unemployed	2 (4.7)	0 (0)	5.95	0.064	2 (8.0)	0 (0)	6.46	0.040	2 (2.2)
Skilled worker	28 (65.1)	42 (84.0)			16 (64.0)	54 (79.4)			70 (75.3)
Professional	13 (30.2)	8 (16.0)			7 (28.0)	14 (20.6)			21 (22.6)
Mother’s occupation									
Housewife	32 (74.4)	39 (73.6)	0.009	0.926	17 (68.0)	54 (76.1)	0.623	0.430	71 (74.0)
Employed/working at home on wages	11 (25.6)	14 (26.4)			8 (32.0)	17 (23.9)			25 (26.0)
Socioeconomic Status (percentile Index)									
1	2 (4.7)	10 (18.9)	23.8	<0.0004	0 (0)	12 (16.9)	15.78	0.003	12 (12.5)
2	2 (4.7)	8 (15.1)			1 (4.0)	9 (12.7)			10 (10.4)
3	4 (9.3)	16 (30.2)			3 (12.0)	17 (23.9)			20 (20.8)
4	12 (27.9)	12 (22.6)			6 (24.0)	18 (25.4)			24 (25.0)
5	23 (53.5)	7 (13.2)			15 (60.0)	15 (21.1)			30 (31.3)
Food insecuity									
Food secure households	37 (86.0)	31 (58.5)	8.72	0.003	23 (92.0)	45 (63.4)	7.33	0.009	68 (70.8)
Food insecure households	6 (14.0)	22 (41.5)			2 (8.0)	26 (36.6)			28 (29.2)
IQ (Mean, SD)	78.8, 7.4	76.2, 5.7	3.82	0.054	82.24, 6.98	75.62, 5.56	22.61	<0.0001	77.4, 6.62
Home environment (Mean, SD)	34.26, 7.5	29.96, 6.6	8.92	0.004	35.7, 8.3	30.55, 6.45	10.03	0.002	31.9, 7.29

*p<0.001.

Data presented as mean ± sd and n (%). Socioeconomic and household food security data was missing for 1 child.

**Fig 2:**
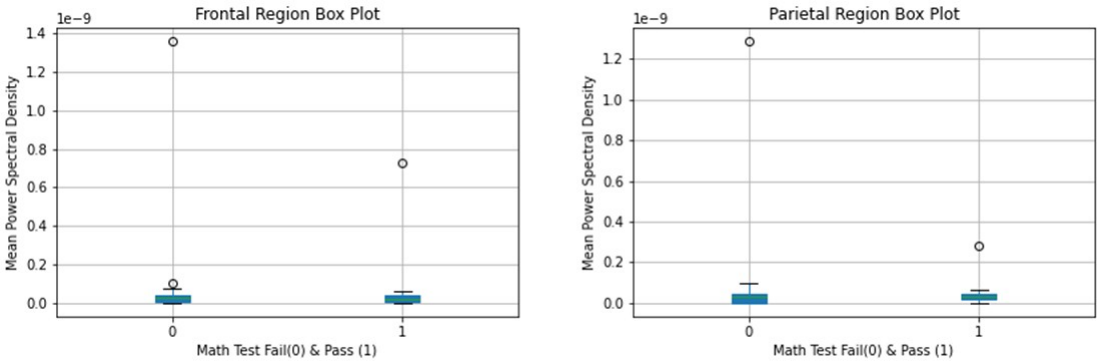
Mean gamma power spectral density by pass vs fail on math test.

**Fig 3:**
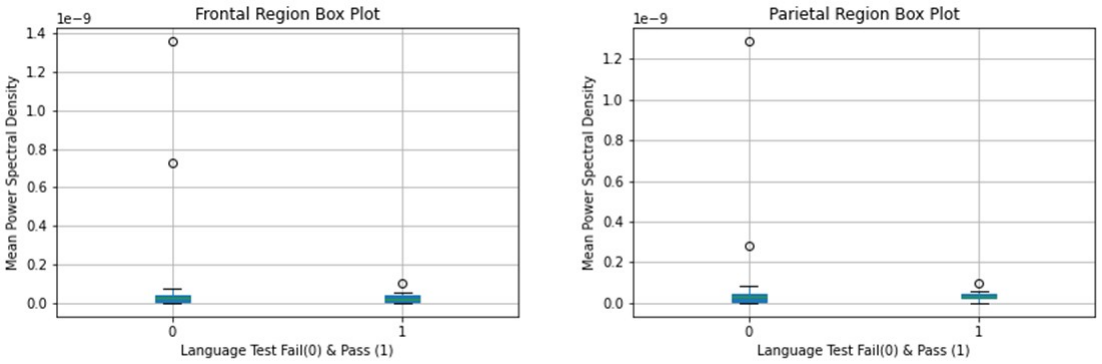
Mean gamma power spectral density by pass vs fail on language test.

Mean gamma power spectral density was calculated for the groups using the above methodology [Figs 2 and 3].

The four different datasets according to the different model combinations were combined with math pass and fail labels as well as language pass and fail labels separately. This resulted in eight different experiments the results are shown in Table 3. The results indicated that sensitivity of EEG only model was similar to the model with addition of SES and home environment variables for Math (58.7% and 57.8%), Language (66.3% and 62.1%) respectively but specificity improved for EEG model with addition of sociodemographic variables for both scores: Math (from 43.6% to 50.6%) and language (from 32% to 60%). The models with IQ scores and sociodemographic scores had greater sensitivity (64.4% and 67.8%) and specificity (55.6% and 76%) compared to the EEG models for Math and language respectively.

**Table 3:** Mean testing data results of KNN using stratified 5-fold cross validation.

Model Combination	Sensitivity	Specificity
EEG *(l = mp vs mf)*	58.7%	43.6%
Sociodemographic + Home Environment + EEG *(l = mp vs mf)*	57.8%	50.6%
EEG *(l = lp vs lf)*	66.3%	32.0%
Sociodemographic + Home Environment + EEG *(l = lp vs lf)*	62.1%	60.0%
IQ *(l = mp vs mf)*	65.8%	46.4%
Sociodemographic + Home Environment + IQ *(l = mp vs mf)*	64.4%	55.6%
IQ *(l = lp vs lf)*	81.5%	48.0%
Sociodemographic + Home Environment + IQ *(l = lp vs lf)*	67.8%	76.0%

Note: *l* denotes the label data, *mp* and *mf* denotes the mathematics test pass and mathematics test fail and *lp* and *lf* denotes the language test pass and language test fail, respectively.

## Discussion

The objective of this study was to use ML techniques as a tool to examine the power of EEG data to predict failure in early years of school. The study found that the EEG data alone were not sufficient to use as a screening tool given very low specificity. However, its combination with sociodemographic and home environment variables considerably increased the specificity. A similar pattern was observed with the models using IQ score as a feature. One reason could be class imbalance that resulted in a low predictive accuracy [31]. While the findings may indicate the EEG alone may not be an adequate screening tool, further validation to examine the predictive power of the combined features model with minimal loss of validity compared to standardized IQ tests may be an interesting avenue to explore.

Factors that predict school performance have been an area of key interest for educationists and policy makers [32]. In order to improve outcomes of school performance, identification of high-risk students is critical so that appropriate interventions can be planned to decrease school dropout rate. In this regard, several standardized intelligence tests have been used to assess physical, cognitive, communication, social, emotional, and/or adaptive development in children mostly developed and normed in the US. A significant effort is required to not just adapt the test culturally but also run psychometric analyses to interpret the scores [33]. The test for IQ scores used the WPPSI III [34] and went through extensive adaptation efforts [35]. Another challenge is that although useful, these measures of cognition for preschoolers have shown limited predictive validity regarding academic performance [34]. This can be a major limitation for use of these cognitive tests in low resource settings. An EEG can also be as resource intensive as the recording also takes at least 20 minutes and a lot of post-processing time. However, the advantage is that the EEG could be a more objective biomarker then tests because the psychological tests might be biased by human factors like subjectivity and induce bias of the assessor or compliance and motivation at the level of the child whereas rest-EEG is only biased by artefacts. Additionally, we believe automation of EEG interpretation using artificial intelligence will be a reality soon thus alleviating the need of highly skilled individuals to interpret EEG waveform abnormalities.

Predicting students’ academic performance at an early age is critical as appropriate interventions can be designed for those who may be struggling with school requirements especially when the government has competing priorities for resource allocation. ML algorithms have been used in the past to predict educational performance of students. Marquez-Vera et al reported that using scores of humanities, language and mathematics, their algorithm was able to accurately identify 98.7% of high school student failures [36]. Ibrahim et al used the students’ demographic profile and the grade point average for the first semester of the undergraduate studies to predict students’ academic performance in the enrolled program and found that ANN appeared to be the best approach to predict the outcome with an accuracy of 80% [37]. Similar work has been done on university students where pre-university and/or first semester grade point average was used to accurately identify 80% of freshmen dropout [38]. It is important to note that all of this work has been done on high school or college students in high income countries and used school grade scores for prediction. A few studies in North America and China have looked at correlations between EEG and neuropsychological or academic status of younger children indicating EEG spectrum to be sensitive to attention-deficit hyperactive disorder [39,40] and predictive of emergent math skills in preschoolers in another study [41]. To the best of our knowledge, our study is the first in a developing country to use EEG data collected on children at the age of 4 years to use ML to predict early school failure in early primary grades.

Under 5 children in developing countries are exposed to multiple risks, including poverty, malnutrition, and un-conducive home environments, which affect their cognitive development [42]. Children not meeting their developmental potential are not well prepared for school with a high risk of failure in early grades and subsequent school drop-out. The proposed model has huge implications for the millions of children who may be at risk of failure in early grades and subsequent drop-out of school. This early development period may identify a unique window of opportunity to intervene early and hence ensure that human capital is used to its utmost potential. These interventions include, but may not be limited to, counseling and family support regarding maximizing the child’s developmental potential, one-on-one attention, and individual educational plans suited for the child’s unique pace and learning abilities [43].

In Pakistan, a large portion of children who do not attain primary education are from rural parts of the country where interventions and screening are limited. In far flung and resource constrained areas, EEG administration by community health workers (CHWs) with decision making by the AI model may assist in screening all pre-primary school age children in the region. Once identified, these high-risk children can receive intervention through trained early childhood educators also enhancing much needed coordination between health and education sectors to help vulnerable children achieve optimal early childhood development outcomes.

This study has strengths and limitations. To the best of our knowledge, there are no existing published studies utilizing ML algorithms that have been used to assess failure in early school grades especially in LMICs. A major limitation of our study was our small population size with an imbalance of the classes especially the language test scores where about two-thirds of children had failed. Though techniques like SMOTE were adopted to deal with this unbalanced data but such techniques do not alleviate the risk of overfitting or bias of the model towards the dominant class. Apart from the small population size the loss of a major proportion of data due to poor quality of data and complete case analysis approach to handle missing data for drawing inferences from the study is also a limitation. ML models improve in accuracy and predictive capability as the size of the data pool increases. However, the current study was a proof of concept and a larger study with prospective data collection specifically targeting the question of validating the algorithm is needed. Further, EEG waves need to be fairly clean data for use in ML which may be a challenge in real world situations, especially in resource constrained settings.

The findings of this study may not be generalized to other countries with low or no failure rate for primary school students unlike Pakistan. Despite its limitations, there is potential of EEG in combination with other variables to predict early grade failure allowing early targeted intervention for highrisk children. In the future, AI system needs to incorporate functional imaging findings that can be applied on children along with EEG findings. The neuroimaging technique is known as functional near infrared spectroscopy (fNIRS) which can be applied on children [44]. fNIRS was used in neurobiological feedback in training that could translate to better educational outcomes such as measures of learning curve by Khoe et al., 2020 [45]. Furthermore, fNIRS can assess hemodynamic changes in the brain when a subject performs cognitive tasks [46].
