# Correction: Use of artificial intelligence on Electroencephalogram (EEG) waveforms to predict failure in early school grades in children from a rural cohort in Pakistan

**DOI:** 10.1371/journal.pone.0247744

**Published:** 2021-02-23

**Authors:** The PLOS ONE Staff

## Notice of republication

This article was republished on February 11, 2021, to remove Supporting Information files that were incorrectly included in the originally published article. The publisher apologizes for the error. Please download this article again to view the correct version.
